# Linear Residual Network Modeling for Anti-HIV-1 Activity Prediction and Docking-Validated Design of Biphenyl-DAPY-Based NNRTIs

**DOI:** 10.3390/molecules31152568

**Published:** 2026-07-23

**Authors:** Huazhao Wang, Yuanyang Zhang, An Wang, Peijian Zhang

**Affiliations:** 1 College of Computer Science and Technology, Qingdao University, Qingdao 266071, China; wanghuazhao618@gmail.com; 2 College of Mathematics and Statistics, Qingdao University, Qingdao 266071, China; yuanyang155@gmail.com (Y.Z.); an02280921@163.com (A.W.)

**Keywords:** Anti-HIV-1 agents, Biphenyl-DAPY, LRNet, rational drug design, molecular dynamics, QSAR

## Abstract

To predict the anti-HIV-1 activity of biphenyl-DAPY-based non-nucleoside reverse transcriptase inhibitors (NNRTIs), a quantitative structure–activity relationship (QSAR) analysis was conducted. Using the heuristic method (HM) for descriptor selection, four predictive models were established: support vector regression (SVR), kernel ridge regression, linear mixed-kernel SVR, and Linear Residual Network (LRNet). Rigorous validations, including leave-one-out cross-validation, fivefold cross-validation, and Y-randomization tests, confirmed their reliability. The LRNet model exhibited the best performance, achieving an average training set $R^{2}$ of 0.8838±0.0090 and an average test set $R^{2}$ of 0.9026±0.0187 over 50 random train–test splits, with $Q_{5-fold}^{2}$ and $Q_{LOO}^{2}$ being 0.8533 and 0.8541, respectively. To further verify the robustness and generalizability of LRNet, independent validation was performed using an external dataset, where LRNet also achieved better generalization performance. The HM and LRNet models were employed to guide the design of novel compounds. Their favorable binding modes with the **1RT2** protein and pharmacokinetic properties were verified via molecular docking and in silico ADMET profiling, respectively. Furthermore, 100 ns molecular dynamics simulations demonstrated the robust dynamic stability, structural compactness, and thermodynamic convergence of the designed candidate within the **1RT2** binding pocket. This study provides a useful computational framework for the rational design and activity prediction of biphenyl-DAPY-based NNRTIs.

## 1. Introduction

Acquired immunodeficiency syndrome (AIDS), caused by human immunodeficiency virus type 1 (HIV-1), remains a severe global public health challenge. The advent of combination antiretroviral therapy (cART) has transformed HIV-1 infection into a manageable chronic disease by suppressing viral replication and preventing disease progression [1]. As cornerstones of cART, non-nucleoside reverse transcriptase inhibitors (NNRTIs) are widely used in clinical practice. They do not require phosphorylation for activation and bind noncompetitively to an allosteric site on HIV-1 reverse transcriptase, inducing conformational changes that inhibit catalytic activity and block reverse transcription of viral RNA [2].

However, the clinical use of existing NNRTIs faces significant challenges. Traditional NNRTI subclasses (e.g., nevirapine, efavirenz, and delavirdine) are particularly prone to inducing drug-resistant mutations [3]. Additionally, adverse effects such as allergic reactions, hepatotoxicity, and high cytotoxicity compromise safety and patient compliance [4]. Consequently, developing novel, highly effective NNRTIs is urgently needed.

Among NNRTI subclasses, biphenyl-diarylpyrimidine (biphenyl-DAPY) derivatives have garnered significant attention in anti-HIV drug development [5]. Incorporating biphenyl substituents onto the DAPY core scaffold enhances hydrophobic and hydrogen-bonding interactions with the HIV-1 reverse transcriptase allosteric site [6]. Nevertheless, the traditional development of these compounds relies heavily on extensive experimental screening. For instance, determining the half-maximal effective concentration (${\mathrm{EC}}_{50}$) in MT-4 cells typically requires the 3-(4,5-dimethylthiazol-2-yl)-2,5-diphenyltetrazolium bromide (MTT) assay [7]. However, these experimental approaches are labor-intensive and time-consuming. Therefore, more efficient methods are needed to predict compound activity and guide rational drug design.

**Figure 1:**
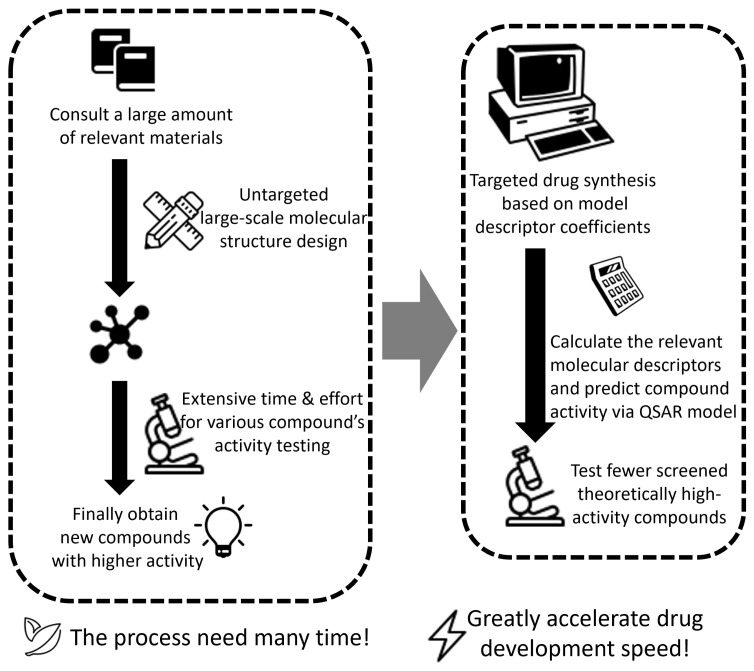
Comparison between traditional R&D methods and the R&D process driven by QSAR.

Quantitative structure–activity relationship (QSAR) modeling is a powerful computational strategy based on the premise that the biological activity of a compound is a function of its molecular structure and physicochemical properties [8]. To establish this relationship computationally, abstract molecular structures are converted into numerical representations known as molecular descriptors, covering multiple dimensions such as molecular topological structure, physicochemical properties, and electronic properties. Robust models that correlate these descriptors with biological activities can, after rigorous validation, reliably and cost-effectively predict the activity of novel molecules. Consequently, this approach facilitates rational drug design and significantly accelerates the drug discovery process (Figure 1).

This study aimed to construct a series of robust QSAR models to predict the anti-HIV-1 activity (${\mathrm{EC}}_{50}$ values) of biphenyl-DAPY-based NNRTIs. Five models of varying complexity were established in the study: a linear regression model developed by the Heuristic Method(HM) for both descriptor screening and model construction, a support vector regression model based on radial basis function kernel (RBF-SVR), a kernel ridge regression model (KRR), linear weighted mixed kernel-based support vector regression (LMIX-SVR), and a Linear Residual Network model (LRNet). All models were evaluated for performance through internal and external validation, in particular, Y-randomization test were implemented for all models to exclude spurious correlations and ensure prediction reliability [9]. Using the HM and LRNet model, derivatives were constructed with the existing compound **M44** (Table 1) as the lead structure. Molecular docking experiments were performed between the screened candidate molecules and the **1RT2** protein [10]. Finally, ADMET profiling was conducted to evaluate the drug-likeness properties of the candidate compounds. This study may provide assistance for the targeted design and activity prediction of subsequent biphenyl-DAPY-based NNRTIs.

**Table 1:** Details of existing compounds.

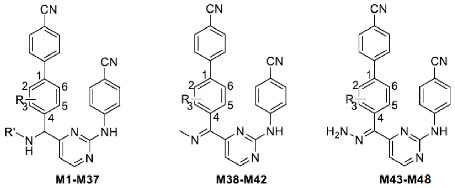				
Compound	R	R′	Measured ${\mathrm{EC}}_{50}$ (μM) ^a^	Predicted by HM
**M1**	3-Me	Me	0.033 ± 0.009	0.0499
**M2**	3-Me	Et	0.063 ± 0.020	0.0651
**M3**	3-Me	n-propyl	0.146 ± 0.055	0.2429
**M4**	3-Me	i-propyl	0.166 ± 0.057	0.1999
**M5**	3-Me	cyclopropyl	0.156 ± 0.055	0.1440
**M6**	3-OMe	Me	0.036 ± 0.011	0.0403
**M7**	3-OMe	Et	0.326 ± 0.282	0.1438
**M8**	3-OMe	n-propyl	0.131 ± 0.032	0.2210
**M9**	3-OMe	i-propyl	0.211 ± 0.084	0.1627
**M10**	3-OMe	cyclopropyl	0.072 ± 0.013	0.0795
**M11**	${\mathrm{3-CF}}_{3}$	Me	0.039 ± 0.014	0.0969
**M12**	${\mathrm{3-CF}}_{3}$	Et	0.153 ± 0.048	0.4540
**M13**	${\mathrm{3-CF}}_{3}$	n-propyl	1.01 ± 0.449	0.9397
**M14**	${\mathrm{3-CF}}_{3}$	i-propyl	1.19 ± 0.000	0.7160
**M15**	${\mathrm{3-CF}}_{3}$	cyclopropyl	0.294 ± 0.098	0.5251
**M16**	${\mathrm{3-OCF}}_{3}$	Me	0.056 ± 0.014	0.0869
**M17**	${\mathrm{3-OCF}}_{3}$	Et	0.505 ± 0.272	0.3994
**M18**	${\mathrm{3-OCF}}_{3}$	n-propyl	0.662 ± 0.303	0.8011
**M19**	${\mathrm{3-OCF}}_{3}$	i-propyl	0.927 ± 0.246	0.6278
**M20**	${\mathrm{3-OCF}}_{3}$	cyclopropyl	0.589 ± 0.190	0.4568
**M21**	3-F	Me	0.064 ± 0.037	0.0278
**M22**	3-F	Et	0.357 ± 0.021	0.1397
**M23**	3-F	n-propyl	0.259 ± 0.087	0.2947
**M24**	3-F	i-propyl	0.649 ± 0.130	0.2557
**M25**	3-F	cyclopropyl	0.630 ± 0.217	0.1744
**M26**	3-Cl	Me	0.011 ± 0.005	0.0223
**M27**	3-Cl	Et	0.099 ± 0.058	0.1120
**M28**	3-Cl	n-propyl	0.184 ± 0.040	0.2323
**M29**	3-Cl	i-propyl	0.230 ± 0.042	0.1958
**M30**	3-Cl	cyclopropyl	0.147 ± 0.034	0.1755
**M31**	3-Cl	${\mathrm{-CH}}_{2}{\mathrm{CF}}_{3}$	0.231 ± 0.077	0.2710
**M32**	3-Cl	${\mathrm{-CH}}_{2}{\mathrm{CH}}_{2}$F	0.077 ± 0.033	0.0760
**M33**	3-Cl	${\mathrm{-CH}}_{2}{\mathrm{CH}}_{2}$CN	0.071 ± 0.031	0.0674
**M34**	3-Cl	${\mathrm{-CH}}_{2}{\mathrm{CH}}_{2}$Cl	0.042 ± 0.018	0.0972
**M35**	3-Cl	${\mathrm{-CH}}_{2}{\mathrm{CH}}_{2}$OH	0.092 ± 0.035	0.0360
**M36**	3-Cl	${\mathrm{-CH}}_{2}{\mathrm{CH}}_{2}{\mathrm{CH}}_{2}$Cl	0.195 ± 0.078	0.1192
**M37**	3-Cl	${\mathrm{-CH}}_{2}{\mathrm{CH}}_{2}{\mathrm{CH}}_{2}$OH	0.067 ± 0.030	0.0665
**M38**	3-Me	–	0.012 ± 0.004	0.0182
**M39**	3-OMe	–	0.008 ± 0.004	0.0119
**M40**	${\mathrm{3-CF}}_{3}$	–	0.035 ± 0.021	0.0325
**M41**	${\mathrm{3-OCF}}_{3}$	–	0.013 ± 0.013	0.0307
**M42**	3-Cl	–	0.006 ± 0.002	0.0056
**M43**	3-Me	–	0.007 ± 0.000	0.0100
**M44**	3-OMe	–	0.005 ± 0.002	0.0082
**M45**	${\mathrm{3-OCF}}_{3}$	–	0.040 ± 0.008	0.0291
**M46**	3-Cl	–	0.006 ± 0.001	0.0036
**M47**	3-F	–	0.007 ± 0.001	0.0041
**M48**	3,${\mathrm{5-F}}_{2}$	–	0.009 ± 0.005	0.0075

^a^ EC_50_: half-maximal effective concentration. Source: Data are from Ref. [11].

## 2. Results

### 2.1. Results of Descriptor Selection

**Figure 2:**
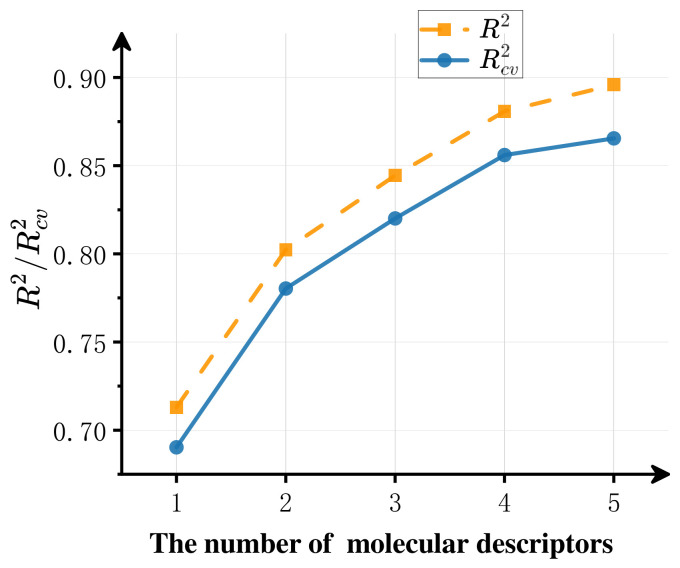
Relationship between $R^{2}$/$R_{cv}^{2}$ and the number of descriptors.

Using CODESSA(v2.63), over 500 molecular descriptors were calculated. Linear models of varying complexity were constructed via the HM by incrementally adding descriptors. The contribution of each descriptor was evaluated by monitoring key statistical parameters ($R^{2}$ and $R_{cv}^{2}$). Figure 2 illustrates these variations. As the number of descriptors increased from one to four, both $R^{2}$ and $R_{cv}^{2}$ exhibited significant, stepwise improvements. However, introducing a fifth descriptor yielded negligible improvement; further increasing the descriptor count risked increasing model complexity and inducing overfitting. Consequently, four descriptors were selected to construct the final linear QSAR model. Based on these four key descriptors, the expression of the constructed HM model is as follows:

(1) $$\begin{array}{cc} -\mathrm{lg}EC_{50}= & -10.940\times \mathrm{RNSB}+1.8138\times (\mathrm{LUMO}-E)\\ \; & -13.038\times \mathrm{MNACH}+23.821\times \mathrm{MPCC}+7.4416 \end{array}$$

MI regression was applied to the 526 molecular descriptors, and the four highest-ranked descriptors were selected for nonlinear modeling. The selected descriptor subset provides a solid foundation for capturing complex nonlinear structure–activity relationships. The features are summarized in Table 2.

### 2.2. Results of HM

**Figure 3:**
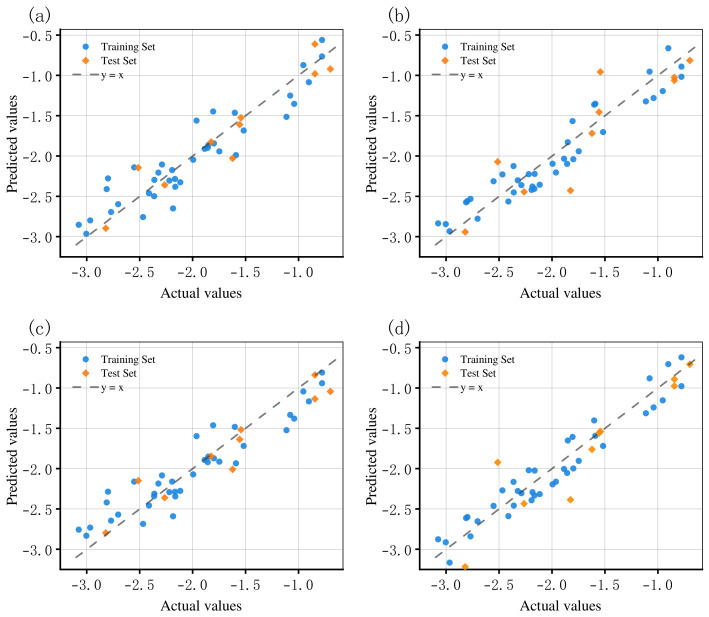
Comparison of predicted vs. experimental ${\mathrm{EC}}_{50}$ values for different QSAR models: (**a**) fitting results of the HM model; (**b**) fitting results of the RBF model; (**c**) fitting results of the KRR model; and (**d**) fitting results of the LMIX-SVR model.

To rigorously evaluate predictive stability, 50 independent random data partitions were employed for all models. Across these 50 runs, the HM model yielded $R_{train}^{2}$ of 0.8691 ± 0.0067 and $R_{test}^{2}$ of 0.8973 ± 0.0181, with $MSE_{train}$ of 0.0553 ± 0.0031, $MSE_{test}$ of 0.0470 ± 0.0126, $Q_{5-fold}^{2}=0.8460$, and $Q_{LOO}^{2}=0.8557$. The average $R^{2}$ values for both the training and test sets exceeded 0.85, indicating that the model can not only fit the linear correlation between descriptors and activity in the training set but also stably predict the activity of compounds in the test set. Meanwhile, $Q_{5-fold}^{2}$ and $Q_{LOO}^{2}$ were similar, confirming that the model maintained stable prediction performance under internal validation. However, as an inherent limitation of linear approaches, the HM model cannot capture potential nonlinear relationships. The results of the nonlinear models will be presented as part of the development of a more comprehensive activity-prediction system. The fitting results for a representative train/test split ($R_{\mathrm{train}}^{2}=0.8639$, $R_{\mathrm{test}}^{2}=0.9073$) are shown in Figure 3a (seed = 44).

Importantly, to ensure a fair and unbiased comparison, the nonlinear models were tested using the dataset containing 4 descriptors selected by the MI method.

### 2.3. Results of RBF-SVR

An RBF-SVR model was constructed based on the non-linear descriptors and the PSO algorithm was used for global optimization. Across the 50 independent random splits, the RBF-SVR model achieved $R_{train}^{2}$ of 0.8511±0.0713, $R_{test}^{2}$ of 0.7248±0.1915, $MSE_{train}$ of 0.0630±0.00304 and $MSE_{test}$ of 0.1226±0.0886. The $Q_{5-fold}^{2}$ is 0.8305, and the $Q_{LOO}^{2}$ is 0.8457.

Compared with the HM modeling method, the training performance of the RBF-SVR model shows comparable fitting capacity. However, the lower average $R_{test}^{2}$ suggests some overfitting due to learning local training noise, which modestly reduced generalization to independent samples. The fitting results for a representative train/test split ($R_{\mathrm{train}}^{2}=0.9073$, $R_{\mathrm{test}}^{2}=0.7765$) are shown in Figure 3b (seed = 44).

### 2.4. Results of KRR

To balance fitting accuracy and generalization performance, a KRR model was subsequently developed. Its performance relies on two key hyperparameters: the RBF kernel coefficient *l* and the regularization strength α.

Across the 50 independent random splits, the KRR model achieved $R_{train}^{2}$ of 0.8481 ± 0.0736, $R_{test}^{2}$ of 0.7654 ± 0.1257, $MSE_{train}$ of 0.0645 ± 0.0316, and $MSE_{test}$ of 0.1045 ± 0.0526. $Q_{5-fold}^{2}$ and $Q_{LOO}^{2}$ are 0.8407 and 0.8447, respectively. These results highlight the critical role of $L_{2}$ regularization in constraining model parameters, thereby controlling model complexity and enhancing generalization. However, due to the limited expressiveness of a single kernel function, the KRR model fails to fully capture the complex nonlinear structure–activity relationships. The fitting results for a representative train/test split ($R_{\mathrm{train}}^{2}=0.7621$, $R_{\mathrm{test}}^{2}=0.7802$) are shown in Figure 3c (seed = 44).

### 2.5. Results of the LMIX-SVR Model

Based on the description in the Methods section, a mixed kernel function was constructed in accordance with Equation (10), and applied to the SVR modeling method to build the LMIX-SVR model. The PSO algorithm was used to optimize the hyperparameters.

The performance indicators of the final LMIX-SVR model are: training set $R^{2}=0.8952\pm 0.0613$, test set $R^{2}=0.8943\pm 0.0582$, $MSE_{train}=0.0445\pm 0.0268$, $MSE_{test}=0.0466\pm 0.0206$, $Q_{5-Fold}^{2}=0.8349$, and $Q_{LOO}^{2}=0.8528$. The difference between the training and test set $R^{2}$ values is much smaller than that observed for KRR, indicating that the introduction of the mixed kernel further improves the model’s ability to capture complex structure–activity relationships and alleviates overfitting. The fitting results for a representative train/test split ($R_{\mathrm{train}}^{2}=0.8891$, $R_{\mathrm{test}}^{2}=0.9141$) are shown in Figure 3d (seed = 44).

### 2.6. Results of the LRNet

As detailed in the Methods section, the LRNet was constructed. Since LRNet was developed based on the HM model, linear descriptors were adopted to evaluate the performance of the LRNet model. The performance indicators of the final LRNet model are as follows: training set $R^{2}=0.8838\pm 0.0090$, test set $R^{2}=0.9026\pm 0.0187$, $MSE_{train}=0.0491\pm 0.0039$, $MSE_{test}=0.0448\pm 0.0134$, $Q_{5-Fold}^{2}=0.8533$, and $Q_{LOO}^{2}=0.8541$. The observed robustness is attributed to the minimalist topology of the network and the introduction of the ReLU6 activation function.

**Figure 4:**
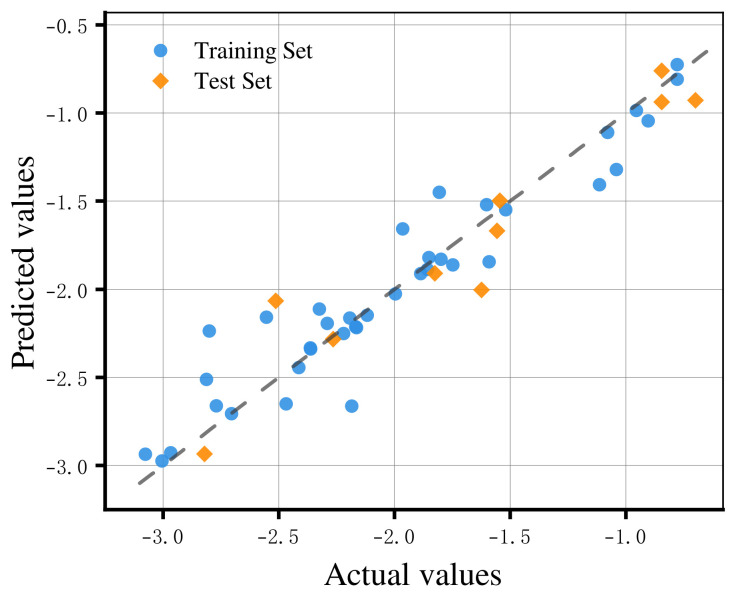
Fitting results of the LRNet model.

The results demonstrate that utilizing a linear mapping as the baseline, complemented by a lightweight neural network for residual correction, is an effective strategy for this specific system. This strategy not only enhances the model’s capacity to capture complex structure–activity relationships but also enables robust generalization on small datasets. Figure 4 illustrates the prediction results of the LRNet model ($R_{train}^{2}=0.8891$, $R_{test}^{2}=0.9141$, seed = 44).

### 2.7. Y-Randomization Results

The Y-randomization test results for the model are shown in Table 3. The extremely low $R_{rand}^{2}$ and $Q_{rand}^{2}$ values indicate that, after disruption of the real structure–activity relationships, the model loses its fitting and predictive capabilities, confirming that it captures the real structure–activity relationship between descriptors and biological activity.

### 2.8. LRNet Robustness and Generalization Validation on an External Dataset

To further evaluate the generalization capability of LRNet on small-sample datasets, an independent and rigorously benchmarked external dataset was selected for validation. Specifically, the dataset compiled by Vinca Prana et al. [12], which comprises 50 nitroaliphatic compounds and their experimental impact sensitivities ($h_{50\%}$), was employed. This dataset has been previously utilized to develop Quantitative Structure–Property Relationship (QSPR) models complying with the strict OECD principles for regulatory acceptability under REACH [12].

In accordance with the original study, the same set of three selected key molecular descriptors was used, and the dataset was partitioned in the same manner, with 34 compounds in the training set and 16 compounds in the external validation set. To perform a rigorous comparative assessment, the LRNet model was evaluated against the validated Multi-Linear Regression (MLR) model established by Prana et al. under identical conditions. The comparative statistical metrics are summarized in Table 4.

As shown in Table 4, the LRNet model demonstrates superior performance across all evaluation metrics. For the training set, a higher goodness-of-fit was achieved by LRNet ($R_{\mathrm{Train}}^{2}=0.9040$, $RMSE_{\mathrm{Train}}=0.1406$) compared to the original MLR model ($R_{\mathrm{Train}}^{2}=0.88$, $RMSE_{\mathrm{Train}}=0.17$). More importantly, on the independent external validation set, LRNet yielded a significantly enhanced predictive accuracy ($R_{\mathrm{EXT}}^{2}=0.8343$, $RMSE_{\mathrm{EXT}}=0.1720$) compared to the benchmark MLR model ($R_{\mathrm{EXT}}^{2}=0.81$, $RMSE_{\mathrm{EXT}}=0.22$). Furthermore, within the defined applicability domain, the internal prediction of LRNet was consistently superior ($R_{\mathrm{IN}}^{2}=0.8073$, $RMSE_{\mathrm{IN}}=0.1776$). The internal robustness of the model was also verified through cross-validation. The cross-validation coefficients of LRNet ($Q_{\mathrm{LOO}}^{2}=0.8511$ and $Q_{5\mathrm{CV}}^{2}=0.8526$) match or exceed those of the original model ($Q_{\mathrm{LOO}}^{2}=0.85$ and $Q_{5\mathrm{CV}}^{2}=0.85$), thereby demonstrating excellent stability.

**Table 4:** Comparison of MLR and LRNet performance.

Metric	Original MLR Model [12]		LRNet Model	
	$R^{2}$	RMSE	$R^{2}$	RMSE
Training Set ($N_{Train}=34$)	0.88	0.17	0.9040	0.1406
External Validation Set ($N_{Ext}=16$)	0.81	0.22	0.8343	0.1720
Internal Validation (Applicability Domain)	0.78	0.23	0.8073	0.1776
$Q_{\mathrm{LOO}}^{2}$	0.85		0.8511	
$Q_{5\mathrm{CV}}^{2}$	0.85		0.8526	

These comparative results further support the rationale for the LRNet architecture. For a small-sample dataset of structurally related compounds with certain linear correlations, the linear branch can capture the overall data trend, while the residual branch further models local nonlinear relationships through the MLP, thereby achieving better prediction accuracy and generalization performance on such datasets.

### 2.9. Design of New Compounds

The average fitted coefficients of the four linear descriptors in the linear branch of LRNet across 50 random splits are −0.4791, 0.2983, −0.1965, and 0.2523, respectively. For comparison, the corresponding average coefficients of the HM are −11.0921, 1.7864, −12.9621, and 23.6802, respectively. Although the absolute magnitudes differ, it is noteworthy that the signs of the corresponding descriptors remain consistent between the two models across the 50 random splits. This consistency provides guidance for the rational design of novel biphenyl-DAPY-based NNRTIs with potentially enhanced activity.

1. RNSB is a structural descriptor that reflects the relative characteristic of the number of single bonds in a molecule, and it can represent structural information such as the saturation of molecular bonds [13].
2. LUMO-E is a quantum chemical descriptor that characterizes a molecule’s ability to accept electrons and is closely related to properties such as electrophilic reactivity [14].
3. MNACH is a descriptor representing the minimum net charge carried by hydrogen atoms in a molecule, reflecting the charge distribution characteristics of hydrogen atoms in the molecule [15].
4. MPCC is a descriptor calculated using the Zefirov method, which represents the minimum partial charge carried by carbon atoms in a molecule and reflects the charge distribution characteristics of these atoms [16].

The negative coefficients of RNSB and MNACH suggest that increased molecular rigidity and enhanced π-conjugation are essential for potent anti-HIV-1 activity [17]. Within the biphenyl-DAPY framework, a reduced proportion of single bonds promotes a robust, planar aromatic system, facilitating stable orientation within the hydrophobic binding pocket [18]. Furthermore, the positive coefficient of LUMO-E indicates that a higher lowest unoccupied molecular orbital energy—which potentially influences the charge-transfer characteristics of the aromatic rings—enhances inhibitory potency [19]. Finally, the positive coefficient of MPCC demonstrates that the electrostatic landscape of the aromatic carbons is a primary determinant of activity. Specifically, increasing the partial positive charge on carbon atoms within the polycyclic system appears to improve binding affinity, likely through strengthened electrostatic or quadrupole interactions with target protein residues [17].

**Figure 5:**
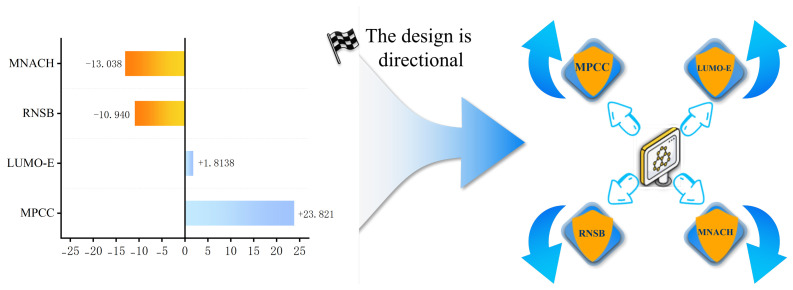
Design of new compounds.

Guided by these theoretical findings, the most active compound, **M44**, was selected as the lead parent scaffold for rational modification, as shown in Figure 5. To strategically modulate the electronic environment of the aromatic rings and enhance the MPCC value, strong electron-withdrawing groups, such as trifluoromethyl (-$CF_{3}$), cyano (-CN), and halogens (-*F*, -Cl), were incorporated at the ortho or para positions relative to the -OMe group, or on the adjacent phenyl ring. These substituents exert potent inductive (-I) and mesomeric (-M) effects, effectively withdrawing π-electron density from the aromatic system. Following these strategies, 94 new compounds were designed.

The newly designed compounds primarily vary in the substituents attached to the terminal phenyl ring. This structural preference is deliberate rather than incidental, as it is strictly guided by the chemical space covered by the training dataset and the model’s AD. Given that the training set predominantly consists of diaryl-DAPY derivatives sharing a common scaffold, this design strategy was intentionally adopted to maximize the likelihood that the novel compounds fall within the model’s AD, thereby ensuring the reliability of the predictive outcomes. Radical structural modifications or scaffold-hopping increase the probability of candidate compounds exceeding the AD warning threshold in terms of leverage value ($h>h^{*}$), thereby rendering the model’s activity predictions for these compounds unreliable.

First, the values of the four key descriptors for all new molecules were calculated using the aforementioned descriptor calculation method. Next, the leverage value $h_{i}$ of each novel molecule was calculated according to the AD analysis method. Subsequently, the value of $h^{*}$ was obtained as 0.3128, and compounds with $h_{i}\leq h^{*}$ were retained. For the remaining compounds, their $-\mathrm{lg}EC_{50}$ values were predicted via the HM and LRNet. Finally, only the compounds whose predicted ${\mathrm{EC}}_{50}$ values from both models were less than 0.005 were retained, and six novel molecules with high predicted activity that met the requirements of the model’s applicability domain were ultimately selected. The chemical structures, predicted ${\mathrm{EC}}_{50}$ values, and leverage values of the candidate molecules are summarized in Table 5.

### 2.10. Molecular Docking Results

The reliability of the Surflex-Dock protocol was validated by redocking the co-crystallized ligand TNK into the NNRTI-binding pocket of **1RT2**. The top-ranked docking pose reproduced the crystallographic binding conformation with a heavy-atom RMSD of 0.2723 Å. The RMSD value was substantially lower than the commonly accepted threshold of 2.0 Å for successful redocking, indicating that the docking protocol was reasonably capable of reproducing the experimentally determined binding pose of the reference ligand. These results provide a reasonable basis for applying the current protocol to the subsequent docking evaluations of the newly designed compounds.

Following the validation of the docking protocol, docking was performed between the candidate molecules and **1RT2**, and docking scores were calculated. The obtained docking scores are shown in Table 5. It can be seen from the table that the average docking score of the candidate molecules is approximately 5.0, which is better than the docking score of compound **M44**. The docking scores of two marketed NNRTIs, Delavirdine (**DLV**) and Efavirenz (**EFV**) [20], were also calculated using the same docking protocol for comparison. **DLV** and **EFV** yielded Total Scores of 7.6370 and 4.8377, respectively. The highest-scoring designed compound, **N1**, achieved a Total Score of 5.7736, which was higher than that of **EFV** but lower than that of **DLV**. These results suggest that **N1** possesses a predicted binding affinity comparable to that of clinically approved NNRTIs.

**Figure 6:**
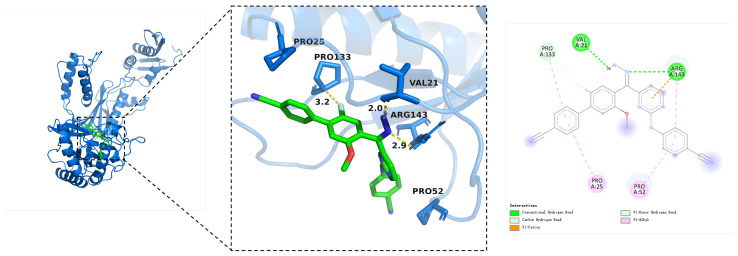
Docking analysis of compound **N1** with **1RT2**.

The PyMOL software (v2.5) was used to visualize the molecular docking results of compounds **N1** and **1RT2**. As illustrated in Figure 6, the biphenyl-DAPY framework of **N1** exhibits a distinct binding orientation, predominantly stabilized by a network of hydrophobic and electrostatic interactions within a specific sub-pocket. A key finding is the significant role of the aromatic substituents in anchoring the ligand. The fluorine-substituted aromatic ring is positioned close to PRO133, with the fluorine atom engaging in a weak *C*-H⋯F contact with the pyrrolidine ring of PRO133 (distance ≈ 3.2 Å). The neighboring aromatic scaffold also participates in hydrophobic π-alkyl interactions with PRO133, supporting ligand stabilization within the binding pocket. Furthermore, the binding conformation is further stabilized by a crucial hydrogen-bonding network. Specifically, the *N*-*H* group of the ligand’s hydrazone linker forms a strong hydrogen bond with the backbone carbonyl of VAL21 (distance ≈ 2.0 Å), while the nitrogen atom interacts via hydrogen bonding with ARG143. Additional hydrophobic π-alkyl interactions with nearby proline residues, including PRO25 and PRO52, further support accommodation of the aromatic scaffold within the binding pocket. Collectively, these interactions may help anchor the biphenyl-DAPY core in a favorable binding orientation and are consistent with the improved docking score of **N1** relative to the lead compound **M44**.

However, a noteworthy limitation in the static docking pose of **N1** is the absence of direct, classical hydrogen-bonding or electrostatic interactions with key residues in **1RT2**, such as Lys101, Lys103, or Tyr181. Since static docking models fail to account for protein flexibility, solvent relaxation, and dynamic induced-fit effects, these results may not fully represent the actual binding capability. To overcome these limitations, evaluate the thermodynamic stability, and assess the synthesis and development potential of **N1** under simulated physiological conditions, a comprehensive 100 ns molecular dynamics (MD) simulation was subsequently performed.

### 2.11. MD Results

To evaluate the dynamic stability, structural compactness, and thermodynamic convergence of **N1** in complex with **1RT2**, a 100 ns molecular dynamics simulation was carried out. Multiple key trajectory-derived metrics, including root-mean-square deviation (RMSD), radius of gyration ($R_{g}$), root-mean-square fluctuation (RMSF), solvent-accessible surface area (SASA), hydrogen-bonding networks, and free energy landscape (FEL), were extracted and systematically analyzed.

**Figure 7:**
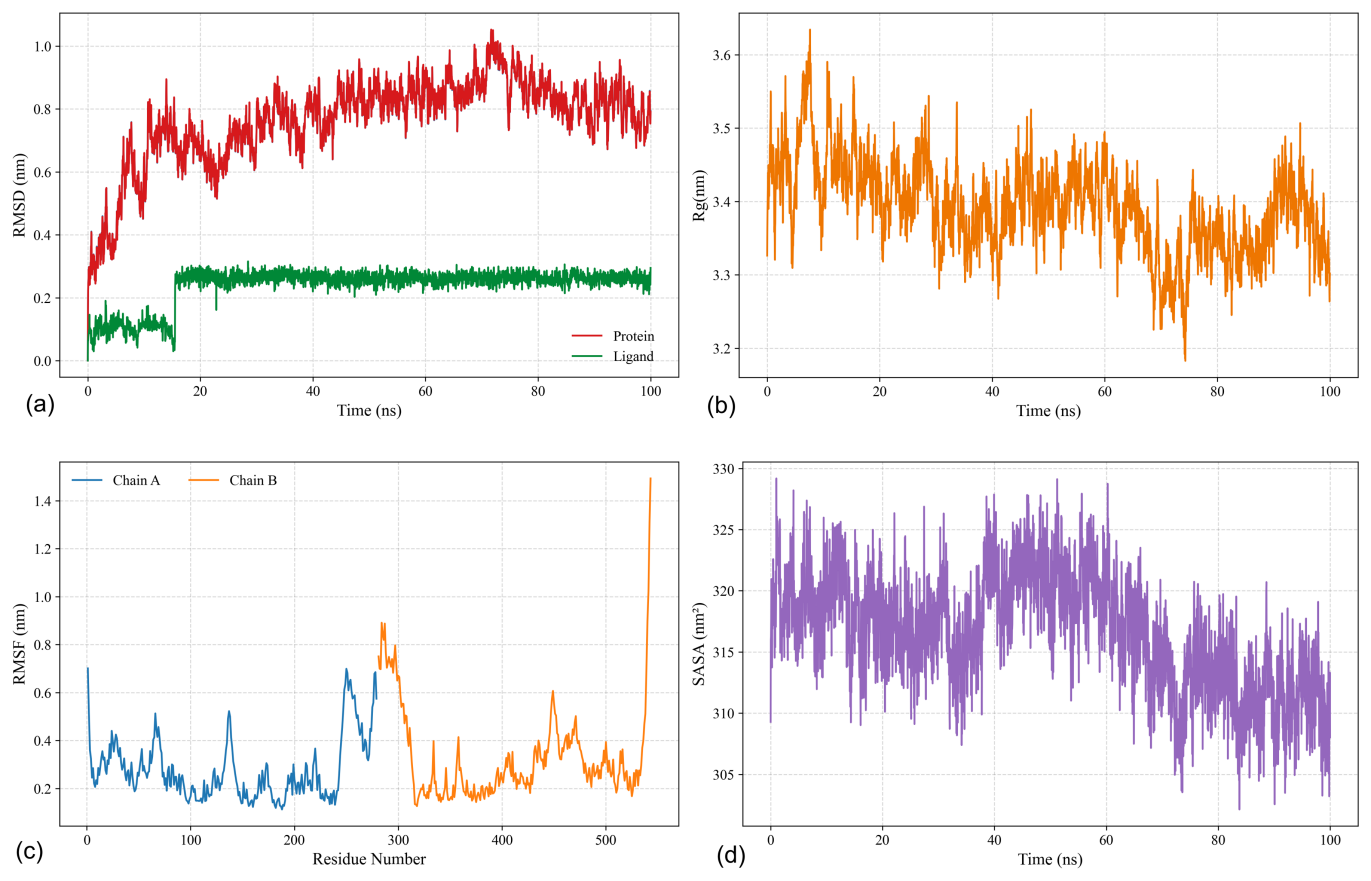
MD analysis of the **N1–1RT2** complex: (**a**) RMSD profiles of the protein (red line) and ligand (green line); (**b**) time evolution of the radius of gyration ($R_{g}$), reflecting the overall structural compactness of the protein; (**c**) RMSF profiles of individual residues in Chain A (blue line) and Chain B (orange line), showing local flexibility; and (**d**) SASA profile of the protein over the 100 ns simulation.

The structural stability of the complex was first assessed using the RMSD profiles of the protein and ligand (Figure 7a). The protein RMSD stabilized at approximately 0.8 nm after initial fluctuations. More importantly, the RMSD of ligand **N1** exhibited a distinct “two-stage” behavior. During the first 15 ns, the ligand remained at a relatively low RMSD matching its initial docked pose. At approximately 15 ns, a sharp, step-like transition to  0.26 nm was observed, after which the curve became remarkably flat and exhibited minimal fluctuations until the end of the 100 ns simulation. This transition indicates a rapid conformational adaptation or induced-fit process wherein the ligand dynamically adjusted its orientation to find a more favorable binding state. The absence of subsequent fluctuations suggests that once this stable conformation was achieved, **N1** remained firmly anchored within the pocket without any detachment.

This structural stabilization is further substantiated by the radius of gyration ($R_{g}$), which reflects the overall compactness of the protein (Figure 7b). The $R_{g}$ value gradually decreased from its initial state (3.45–3.50 nm) and converged to approximately 3.35 nm in the late stages of the simulation. This downward trend suggests that binding of **N1** may be associated with increased structural compactness of the protein.

The local flexibility of the receptor was examined using the RMSF of individual residues across Chain A and Chain B (Figure 7c). Highly symmetrical fluctuation patterns were observed for both chains. The core regions of the protein, particularly those surrounding the binding pocket, maintained low fluctuations with RMSF values well below 0.25 nm, indicating that the active site is structurally well-preserved. In contrast, high fluctuations were restricted to the terminal regions (especially the C-terminus of Chain B, which peaked at 1.5 nm). This localized terminal flexibility explains the relatively high overall protein RMSD (0.8 nm) while the ligand-binding core remained rigid. The overall structural compactness of the protein is further supported by the SASA profile (Figure 7d), where the surface area decreased from approximately $320{\mathrm{nm}}^{2}$ to a relatively stable range of approximately $310{\mathrm{nm}}^{2}$ after 60 ns. This reduction is consistent with decreased solvent exposure during the later stages of the simulation.

**Figure 8:**
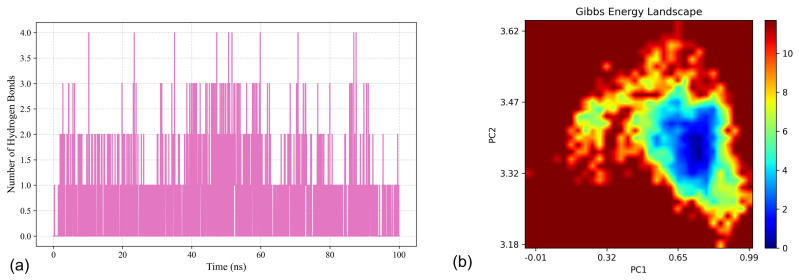
Interaction networks and conformational free-energy landscape of the **N1**–**1RT2** complex: (**a**) Temporal evolution of the number of intermolecular hydrogen bonds formed between **N1** and the protein during the 100 ns molecular dynamics simulation; and (**b**) two-dimensional free-energy landscape projected onto PC1 and PC2, with PC2 corresponding to the radius of gyration ($R_{g}$).

To elucidate the driving forces behind this dynamic stability, intermolecular hydrogen bonding between **N1** and the protein was analyzed (Figure 8a). Despite the lack of classical key-site polar interactions in the initial static docking, the dynamic simulation revealed a highly resilient polar interaction network. On average, 1 to 2 hydrogen bonds were dynamically maintained throughout the trajectory, with transient peaks reaching up to 3 or 4 bonds. Notably, the frequency of maintaining multiple hydrogen bonds increased after the conformational adjustment at 15 ns, indicating that the ligand’s adaptation successfully optimized its orientation to engage in more stable polar contacts with neighboring residues.

The thermodynamic stability of the complex was finally mapped using the two-dimensional free energy landscape (FEL), where the first principal component (PC1) and the second principal component (PC2, corresponding to $R_{g}$) served as reaction coordinates (Figure 8b). The FEL depicts a single, highly concentrated, and well-defined global energy minimum basin (Gibbs free energy of 0 kJ/mol) centered at PC1 ≈ 0.65–0.75 nm and PC2 ≈ 3.32–3.42 nm. The presence of this dominant and well-defined energy basin supports good thermodynamic convergence of the simulated system. It demonstrates that the transition at 15 ns represented the relaxation of the system into its global thermodynamic minimum.

In conclusion, although static molecular docking suggested a lack of direct interactions between **N1** and classical active residues, the dynamic simulation successfully demonstrated that **N1** can dynamically adapt its conformation to establish a highly stable, compact, and thermodynamically converged complex with HIV-1 RT. Supported by dynamic hydrogen bonds and enhanced hydrophobic shielding, the robust dynamic stability of **N1** further supports its potential as a promising lead candidate for further drug development.

### 2.12. ADMET Profiling

To assess the translational potential of the designed candidates, their physicochemical properties and ADMET profiles were evaluated. Crucially, these properties were benchmarked against four established drug-likeness and bioavailability filters: the Lipinski, Veber, Ghose, and Egan rules [21].

As shown in Table 6, the lipophilicity (log *P*) values for the designed compounds range from 3.885 to 5.06. While most candidates maintain an optimal lipophilicity range, compound **N4** exhibits a logP of 5.06, which marginally exceeds the Lipinski threshold (log *P* ≤ 5).

Furthermore, the molecular weights (MWs) of compounds **N3** (513.09), **N4** (549.13), and **N5** (536.18) exceed 500 Da. High molecular weight and elevated lipophilicity are known to reduce aqueous solubility and oral bioavailability, which is reflected in the lower Quantitative Estimate of Drug-likeness (QED) score for **N4** (0.126). Under Lipinski’s Rule of 5 (which permits up to one violation), **N3** remains compliant (one violation: MW), whereas **N4** fails due to two violations (MW and logP). In terms of the Ghose filter (which restricts MW to 160–480 and logP to −0.4 to 5.6), only the low-MW compounds (**M44**, **N1**, **N2**, and **N6**) strictly comply, while **N3** and **N4** violate the upper MW limit.

In terms of polar surface area and absorption, the majority of the compounds (**M44**, **N1**–**N4**) exhibit a Topological Polar Surface Area (TPSA) of $133.00\;{Å}^{2}$. Assuming acceptable numbers of rotatable bonds and TPSA values within the Veber threshold (TPSA $\leq 140\;{Å}^{2}$), these candidates are predicted to possess acceptable oral bioavailability. However, a TPSA of $133.00\;{Å}^{2}$ marginally exceeds the Egan filter limit (TPSA $\leq 131.6\;{Å}^{2}$), suggesting a borderline threshold for membrane permeability and passive absorption. In contrast, compound **N6** (TPSA = $156.79\;{Å}^{2}$) and the excluded compound **N5** (TPSA = $192.94\;{Å}^{2}$) fail both the Veber and Egan filters, highlighting a high risk of poor membrane permeability.

While all compounds are predicted to have high human intestinal absorption (HIA), safety screening via the AMES toxicity predictor revealed that **N5** is AMES-positive, indicating mutagenic potential. Coupled with its unfavorable TPSA, **N5** was excluded from further drug design stages. The remaining candidates, particularly **N1** and **N2**, demonstrate highly balanced profiles, showing no Lipinski or Veber violations and only a marginal Egan TPSA exceedance, suggesting relatively favorable drug-like properties.

## 3. Discussion

Table 7 presents a comparative analysis of the activity prediction performance of multiple QSAR models in this study. It can be observed that different models exhibit distinct performance characteristics: The HM model passes the Y-randomization test, indicating good predictive performance, but it cannot capture nonlinear correlations in structure–activity relationships. The RBF-SVR model can capture nonlinear structure–activity correlations by relying on the strong learning ability of the RBF kernel, but it suffers from overfitting due to the inherent characteristics of the kernel function. The KRR model improves overfitting through $L_{2}$ regularization, leading to enhanced generalization stability. However, the single RBF kernel has limited expressive ability, resulting in insufficient exploration of complex structure–activity relationships. After introducing the mixed kernel function, the LMIX-SVR model overcomes the limitations of single kernels and further balances fitting accuracy and generalization ability. Ultimately, LRNet emerges as the optimal model in this study. By innovatively integrating a linear baseline with a lightweight neural network for residual correction, it successfully synergizes the physical interpretability of linear models with the powerful representation capacity of non-linear algorithms. Overall, all constructed models demonstrate good predictive performance and provide valuable guidance for the structural optimization of biphenyl-DAPY-based NNRTIs.

However, this study still has the following limitations:

- The dataset size is relatively small, which may limit the generalization capability of the models for structurally diverse biphenyl-DAPY derivatives and make it difficult to cover broader structure–activity relationship space within this class of compounds.
- The AD established by the HM is relatively narrow and predominantly comprises biphenyl-DAPY derivatives sharing a common core scaffold. Consequently, the newly designed compounds remain highly conservative within the chemical space, with structural variations largely confined to the optimization of aromatic substituents. This constraint inherently limits the framework’s capacity to discover structurally diverse candidates and may also result in some compounds with novel structural fragments being excluded from the applicability domain.

Based on the above limitations, future research can be advanced in two directions:

1. Expand the dataset size by incorporating more biphenyl-DAPY derivatives with different substituents and activity levels to further enrich structure–activity relationship information and broaden the generalization boundary of the models.
2. Broaden the applicability domain of the HM by incorporating a larger and more structurally diverse dataset containing multiple NNRTI scaffolds, thereby enabling the prediction and generation of compounds with novel scaffolds.

## 4. Materials and Methods

### 4.1. Data Overview

The dataset of biphenyl-DAPY derivatives used in this study was obtained from the literature (Chen et al.) [11], and the chemical structures and activities of the compounds are summarized in Table 1. The dataset consists of two core components: the molecular structures of all biphenyl-DAPY derivatives and their anti-HIV-1 activity values, quantified as ${\mathrm{EC}}_{50}$. All ${\mathrm{EC}}_{50}$ values were measured under uniform experimental conditions (MT-4 cells as the host cell line and MTT colorimetric assay targeting the wild-type HIV-1 IIIB strain). Table 1 shows that the original measured anti-HIV-1 activity values of the compounds are in μM, and most of these values are in the form of extremely small numbers. To avoid potential numerical inaccuracies caused by extremely small concentration values during logarithmic transformation, the original ${\mathrm{EC}}_{50}$ values in μM were first multiplied by 1000 and converted to nM, thereby converting the values into integers or near-integers; subsequently, the negative logarithm of the converted ${\mathrm{EC}}_{50}$ values was calculated.

### 4.2. Descriptor Calculation

Molecular descriptor calculation is a fundamental step in QSAR modeling, because descriptor quality depends strongly on the consistency of the underlying molecular geometries and electronic structures. To ensure reliability and reproducibility, all compounds were processed using the same computational workflow [22].

First, the two-dimensional molecular structures were constructed in ChemDraw (8.0) and saved as SKC files, which were then imported into HyperChem Professional (Release 7) to generate the initial three-dimensional geometries. Because geometries generated from two-dimensional structures may contain unreasonable bond lengths, bond angles, or steric clashes, a two-step geometry optimization protocol was applied. Initial optimization was performed using the MM+ molecular mechanics force field to remove unfavorable structural strain and obtain reasonable starting conformations. The resulting structures were then further refined using the semi-empirical AM1 method to relax the molecules toward local energy minima. This procedure minimized the influence of the initially generated coordinates and ensured that all descriptors were calculated from a standardized set of optimized geometries.

Although this local geometry optimization protocol does not guarantee identification of the absolute global minimum or the experimentally relevant bioactive conformation, it is suitable for descriptor-based QSAR modeling of a congeneric series. The compounds investigated in this study share similar scaffolds and have a limited number of degrees of freedom; therefore, differences in the calculated molecular properties are expected to arise mainly from substituent effects rather than major conformational changes. Moreover, the absolute gas-phase global minimum is not necessarily the bioactive conformation adopted during target binding. Thus, by applying the same 2D-to-3D conversion and local optimization workflow to all compounds, the resulting descriptors can consistently reflect relative variations in structural and electronic properties across the dataset, providing a reliable basis for QSAR modeling [8].

The optimized molecular geometries were then exported from HyperChem in both HIN and ZMT formats. The ZMT files were submitted to MOPAC (MOPAC 6) for semi-empirical quantum-chemical calculations to generate the electronic structure information required by CODESSA (v2.63), producing the corresponding MNO output files [23]. Therefore, MOPAC was used not merely for file conversion but to perform the quantum-chemical calculations required for descriptor generation. Finally, the optimized HIN files, the MOPAC-generated MNO files, and the converted biological activity values were imported into CODESSA to calculate the complete set of molecular descriptors [24].

### 4.3. Descriptor Selection

In this study, the linear descriptor set was selected based on the research by Katritzky et al. [2], using a model that follows the heuristic method implemented in CODESSA. First, preselection is performed on the original descriptor set: constant or approximately constant descriptors, items with excessive missing values, and items with extremely small variance are eliminated; meanwhile, highly redundant descriptors are merged or removed. Subsequently, the cross-correlation between all descriptors is calculated, and orthogonal (or approximately orthogonal) descriptor pairs are identified. Binary regression models are established starting from these orthogonal pairs, and pairs with higher correlation coefficients are selected. For each candidate subset, new non-collinear descriptors are gradually added; the statistics of the model after the addition of each new descriptor are calculated, and the significance increase criterion (e.g., Fisher’s criterion) is used to determine whether to accept the descriptor. In addition to Fisher’s criterion, indicators such as $R^{2}$ and $R_{cv}^{2}$ are comprehensively considered to balance statistical significance and prediction stability. This process ensures that each introduced descriptor can significantly improve the model performance, and the increase in the number of descriptors is stopped when there is no longer a significant gain in the model’s explanatory power, thus effectively controlling the risk of overfitting while ensuring the model’s prediction ability. The selected descriptor values were standardized for the construction of subsequent models. The key descriptor set is shown in Table 8.

To eliminate the potential bias introduced by using descriptors optimized for linear models when developing nonlinear models, an independent nonlinear descriptor selection strategy based on mutual information (MI) regression was adopted in this study. First, the complete set of molecular descriptors was exported from the CODESSA software (version 2.63). However, because the number and types of available descriptors varied among different molecules, the descriptor dimensions were aligned using a zero-padding strategy to construct a unified feature matrix. As a result, an original descriptor dataset containing 526 molecular descriptors was obtained. The original descriptor matrix was subsequently preprocessed before feature selection. Constant descriptors with zero variance were removed to eliminate uninformative features, and the remaining descriptors were normalized using Z-score standardization to minimize scaling bias.

Subsequently, MI [25] regression was employed to rank and select the descriptor subset for the nonlinear models. MI is a nonparametric measure derived from information theory that quantifies the amount of information shared between two variables. Unlike the conventional linear correlation coefficient, MI is capable of capturing arbitrary dependencies, including highly complex nonlinear relationships, without making any assumptions about the underlying data distribution. The mutual information between a molecular descriptor (*X*) and the biological activity (*Y*) (${\mathrm{EC}}_{50}$) is defined as

(2) $$I\left(X;Y\right)=\;\int \int \;p\left(x,y\right)\log\frac{p(x,y)}{p(x)p(y)},dx,dy,$$

where p(x,y) denotes the joint probability density function of *X* and *Y*, while p(x) and p(y) represent their marginal probability density functions, respectively. In practice, MI scores were estimated using a nonparametric entropy estimator based on *k*-nearest neighbor distances [26]. All preprocessed molecular descriptors were ranked according to their estimated MI scores with respect to the biological activity. To ensure a rigorous and unbiased comparison with the linear model, the four descriptors with the highest MI scores were selected to construct the subsequent nonlinear models, thereby maintaining the same feature dimensionality.

### 4.4. Support Vector Regression (SVR)

In QSAR studies, the relationship between molecular structure and biological activity often exhibits nonlinear characteristics; therefore, nonlinear modeling methods are required to enhance prediction performance. Meanwhile, to ensure model consistency and comparability, a unified set of molecular descriptors is employed.

**Figure 9:**
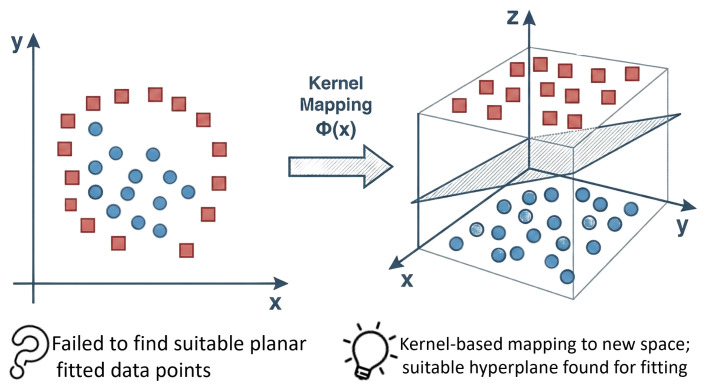
Illustration of Kernel Mapping, where red squares and blue circles represent two different classes of data points.

SVR is a statistical learning-based regression method that seeks an optimal hyperplane such that the prediction errors for most training points lie within a prescribed tolerance ε [27]. As illustrated in Figure 9, SVR may first map the original descriptor vectors into a higher-dimensional feature space via a nonlinear mapping ϕ(x); in that space an appropriate hyperplane can be found even when the original data are not linearly separable.The optimization problem of SVR can be expressed as follows:

(3) $$\min_{w,b,\xi_{i},\xi_{i}^{*}}\frac{1}{2}{∥w∥}^{2}+C\sum_{i=1}^{n}\left(\xi_{i}+\xi_{i}^{*}\right)$$

with the constraints:

$$\left\{\begin{array}{c} y_{i}-\left(w^{T}\phi \left(x_{i}\right)+b\right)\leq \varepsilon +\xi_{i},\\ \left(w^{T}\phi \left(x_{i}\right)+b\right)-y_{i}\leq \varepsilon +\xi_{i}^{*},\\ \xi_{i},\xi_{i}^{*}\geq 0, \end{array}\right.$$

where ε is the threshold of the ε-insensitive loss function, and C>0 is the penalty parameter. By introducing Lagrange multipliers ${\alpha_{i},\alpha_{i}^{*}}_{i=1}^{n}$, the original optimization problem can be transformed into a dual form that is easier to solve. The regression function obtained from the dual solution is:

(4) $$\hat{y}\left(x\right)=\sum_{i=1}^{n}\left(\alpha_{i}-\alpha_{i}^{*}\right)\;\langle \phi \left(x_{i}\right),\phi \left(x\right)\rangle +b,$$

### 4.5. Kernel Functions and Kernel Trick

In the dual formulation of the regression function, ϕ(·) may project the data into a feature space of very high dimensionality, so explicitly computing $\langle \phi \left(x_{i}\right),\phi \left(x_{j}\right)\rangle$ is impractical. The kernel trick evaluates this inner product directly from the original inputs as $k(x_{i},x_{j})$, i.e.,

(5) $$k\left(x_{i},x_{j}\right)=\langle \phi \left(x_{i}\right),\phi \left(x_{j}\right)\rangle$$

Choosing an appropriate kernel is essential for SVR to capture nonlinear relationships effectively. The validity of kernel functions is guaranteed by Mercer’s Theorem: If a kernel function k(x,y) is a continuous symmetric function defined on the sample space, and for any square-integrable function g(·), the following condition is satisfied:

(6) ∫∫k(x,y)g(x)g(y)dxdy≥0

i.e., there exists a corresponding mapping function ϕ(·) such that $k\left(x,y\right)=\langle \phi (x),\phi (y)\rangle$.

In addition, Mercer’s Theorem also implies several important closure properties of kernel functions: If both $k_{1}$ and $k_{2}$ are valid kernel functions, then: the product of a kernel function and a constant ($\alpha \times k_{1},\;\alpha \geq 0$) is still a kernel function; their non-negative linear combination $\alpha k_{1}+\beta k_{2}$ (α,β≥0) is also a kernel function.

Commonly used kernel functions in SVR include the radial basis function (RBF) kernel and the linear kernel [28]. The RBF kernel is widely adopted due to its ability to flexibly characterize complex nonlinear relationships. Its expression is:

(7) $$k_{\mathrm{RBF}}\left(x_{i},x_{j}\right)=\exp\left(-\frac{|x_{i}-x_{j}{|}^{2}}{2l^{2}}\right)$$

where *l* is the length scale parameter, which is used to control the smoothness of the kernel function. The linear kernel is another commonly used kernel function in SVR, especially suitable for problems where the relationship between input features and the target variable is approximately linear. Its expression is given by

(8) $$k_{\mathrm{Linear}}\left(x_{i},x_{j}\right)={x_{i}}^{⊤}x_{j}$$

### 4.6. RBF-SVR Method

The RBF-SVR method was proposed in accordance with Equation (7). Although the RBF kernel exhibits strong learning ability for training data due to its flexible nonlinear mapping capability and can capture subtle structure–activity relationship patterns, this flexibility may also cause the model to overfit noise in the training set, thereby impairing its generalization ability [29]. To address this issue, the kernel ridge regression (KRR) modeling method and the mixed kernel method were introduced.

### 4.7. KRR Method

KRR combines the advantages of ridge regression and kernel methods [30]. Its core idea is to map the original descriptor space to a high-dimensional feature space via a kernel function, then perform ridge regression. By integrating the $L_{2}$ regularization of ridge regression with the kernel trick, KRR not only enables nonlinear fitting but also suppresses overfitting through regularization. The objective function of KRR is defined as follows:

(9) $$\min_{w}\left\{\frac{1}{n}\sum_{i=1}^{n}{\left(y_{i}-w^{T}\phi \left(x_{i}\right)\right)}^{2}\right\}+\alpha {∥w∥}^{2}$$

where α>0 is the regularization parameter. The regularization term $\alpha {∥w∥}^{2}$ in the equation reduces model complexity by penalizing the magnitude of the weight vector, thereby alleviating the overfitting problem of the model. The RBF kernel was still chosen for the KRR model.

### 4.8. LMIX-SVR Method

Although the KRR model alleviates the overfitting problem of RBF-SVR to a certain extent, using a single RBF kernel can restrict the model’s fitting capacity and hinder the full exploitation of implicit structure–activity relationships. This study introduced the LMIX-SVR modeling method to integrate the advantages of different kernel functions [31]. The mixed kernel function is defined as follows:

(10) $$k_{mix}\left(x_{i},x_{j}\right)=w_{rbf}\cdot k_{rbf}\left(x_{i},x_{j}\right)+w_{linear}\cdot k_{linear}\left(x_{i},x_{j}\right)$$

Here, $w_{rbf}$ and $w_{linear}$ are non-negative weight coefficients satisfying $w_{rbf}+w_{linear}=1$. By linearly weighting the RBF kernel and linear kernel, the method synergistically leverages the strong local nonlinear mapping capability of the RBF kernel and the global linear representation ability of the linear kernel, allowing the model to more precisely capture both linear trends and nonlinear variations.

### 4.9. LRNet Method

The descriptors selected by the HM show a certain degree of linear correlation with the activity values. However, traditional pure linear models are limited by their own structure and are unable to capture the complex nonlinear relationships. Although non-linear models (e.g., KRR and LMIX-SVR) have strong fitting capabilities, their global nonlinear mappings often lack interpretability and are difficult to directly guide the design of new compounds. Under this research framework, a customized LRNet that aligns with the characteristics of the current dataset was proposed. This model makes predictions based on a linear model and uses a lightweight neural network as the residual branch for nonlinear correction. Specifically, the architecture of LRNet consists of two branches and the final output is the superposition of the two branches:

1. Linear branch: a single linear mapping without activation, intended to directly capture the dominant linear relationship between descriptors and activity.
2. Residual branch: a highly compact multilayer perceptron comprising a single hidden layer with four neurons and a linear output layer, dedicated to modeling the remaining nonlinear signal.

**Figure 10:**
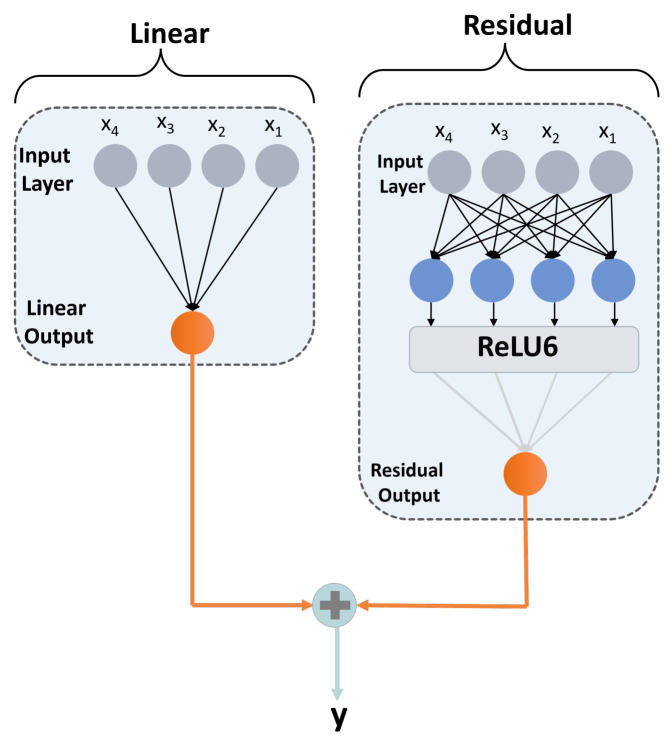
The network structure diagram of LRNet.

Figure 10 presents the network structure diagram of LRNet.

Given the limited sample size in this study, this minimalist network topology can effectively reduce the risk of severe overfitting. Moreover, the hidden layer employs the ReLU6 activation function with an upper bound constraint:

(11) ReLU6(x)=min(max(x,0),6)

Compared to the traditional ReLU, ReLU6 can effectively suppress the extreme activation values that are prone to occur in small datasets, further improving the training stability and generalization ability of the model [32]. Under the current data and descriptor-selection scheme, LRNet demonstrated favorable performance, and its linear branch can be integrated with the HM, providing an intuitive reference for the targeted design of novel compounds.

### 4.10. Particle Swarm Optimization (PSO) Algorithm

In the process of machine learning model construction, the determination of hyperparameters directly affects model performance. Some nonlinear models contain a relatively large number of interacting hyperparameters. However, traditional hyperparameter optimization methods may suffer from efficiency bottlenecks. As a heuristic optimization method based on swarm intelligence [33], the PSO algorithm can efficiently search the hyperparameter space by simulating the collective cooperative behavior of bird flocks during foraging. The central concept of the PSO algorithm is that it treats each potential solution to the optimization problem as a “particle” in the search space. Each particle dynamically adjusts its movement state by tracking its own historical optimal position (pbest) and the global optimal position of the swarm (gbest), gradually approaching the optimal solution.

Assuming the dimension of the search space is *D*, the position of the *i*-th particle is $x_{i}=\left(x_{i1},x_{i2},\ldots ,x_{iD}\right)$, and its velocity is $v_{i}=\left(v_{i1},v_{i2},\ldots ,v_{iD}\right)$. The update formulas for its velocity and position are as follows [34]:

(12) $$\begin{array}{cc} v_{id}\left(t+1\right)=w\cdot v_{id}\left(t\right)+c_{1}\cdot r_{1}\cdot & (pbest_{id}-x_{id}\left(t\right))+c_{2}\cdot r_{2}\cdot \left(gbest_{d}-x_{id}\left(t\right)\right) \end{array}$$

(13) $$\begin{array}{cc} x_{id}\left(t+1\right) & =x_{id}\left(t\right)+v_{id}\left(t+1\right) \end{array}$$

where

1. *t*: iteration number;
2. $c_{1}$, $c_{2}$: acceleration coefficients;
3. $r_{1}$, $r_{2}$: random numbers within the interval 0,1, which increase the randomness of the search.

Through iterative updates, the particle swarm can efficiently approach the optimal solution, realizing efficient search in the hyperparameter space.

### 4.11. Model Validation

Model validation includes internal validation and external validation, which evaluate the model’s internal prediction stability and generalization ability respectively. Internal validation adopts five-fold cross-validation ($Q_{5-fold}^{2}$) and leave-one-out cross-validation ($Q_{LOO}^{2}$); the higher the values of these two indicators, the better the internal predictive performance. External validation refers to evaluating the model’s prediction ability using an independent test set. Common indicators include the coefficient of determination ($R^{2}$), which quantifies the extent to which the variance of observed responses is accounted for by the model’s predicted values. The higher the $R^{2}$, the better the model fit. Mean squared error (MSE) is used to evaluate the model’s prediction accuracy and reflects the mean squared deviation between predicted values and actual activity values. MSE is always non-negative; the smaller its value, the higher the prediction accuracy.

### 4.12. Y-Randomization Test

In QSAR modeling, relying solely on traditional performance metrics (e.g., $R^{2}$) cannot reliably distinguish genuine structure–activity relationships from chance correlations. As a rigorous validation method, the Y-randomization test detects spurious correlations by disrupting the true association between features and the target variable [9].

Specifically, the dependent variable vector *Y* is randomly permuted multiple times to uncouple it from the feature matrix *X*. Models are then reconstructed using the randomized data under identical modeling protocols. If the original model’s performance significantly exceeds that of the randomized models, this confirms the capture of real structure–activity signals; otherwise, the model likely relies on spurious correlations. Two key metrics evaluate the randomized models:

1. Coefficient of determination $R_{rand}^{2}$:
  (14) $$R_{rand}^{2}=1-\frac{\sum {\left(Y_{scrambled}-{\hat{Y}}_{pred}\right)}^{2}}{\sum {\left(Y_{scrambled}-{\bar{Y}}_{scrambled}\right)}^{2}}$$
2. Cross-validation coefficient of determination $Q_{rand}^{2}$:
  (15) $$Q_{rand}^{2}=1-\frac{\sum {\left(Y_{scrambled}-{\hat{Y}}_{cv\_pred}\right)}^{2}}{\sum {\left(Y_{scrambled}-{\bar{Y}}_{scrambled}\right)}^{2}}$$

Repeating the Y-randomization experiment 1000 times ensures robust conclusions. Applying this test to the models further verified their reliability and supported their application in guiding subsequent compound design.

### 4.13. Applicability Domain (AD) Analysis

In QSAR studies, model reliability is inherently domain-dependent; predictions for novel compounds may be inaccurate if they fall outside the modeled chemical space. Therefore, applicability domain (AD) analysis is essential to delineate the region where QSAR predictions are trustworthy [35].

**Figure 11:**
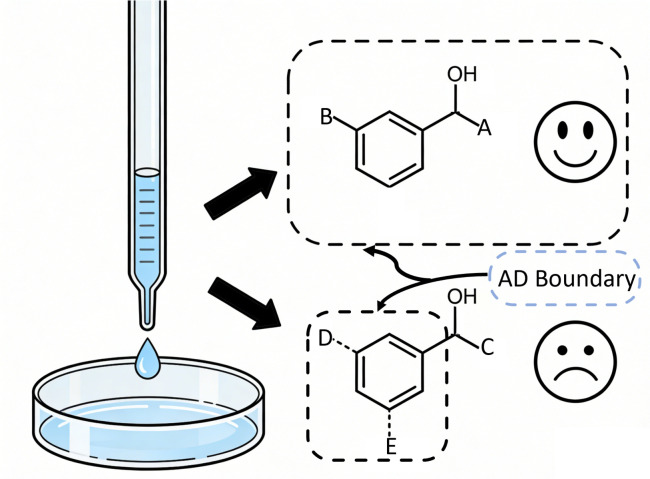
AD analysis to identify the prediction range for novel compounds.

As illustrated in Figure 11, existing compounds collectively define a bounded chemical space within which structurally similar query molecules yield reliable predictions. Conversely, compounds beyond this AD boundary exhibit high prediction uncertainty. To identify this boundary, various approaches have been developed, including distance-, density-, and leverage-based methods. In this study, the AD analysis was performed using the leverage value method to delineate the prediction range for novel compounds. The leverage value method judges whether a sample lies within the AD by quantifying the “similarity” between the sample and the overall dataset, and it includes two core indicators:

1. Leverage value ($h_{i}$): Describes the degree of influence of a single sample on the model and reflects the extent to which its position in the descriptor space deviates from the center of the dataset.
  (16) $$h_{i}=x_{i}{\left(X^{T}X\right)}^{-1}x_{i}^{T}$$
  where $x_{i}$ is the descriptor vector of the *i*-th sample, X is the descriptor matrix of the dataset, and ${\left(X^{T}X\right)}^{-1}$ is the inverse matrix of the covariance matrix of the dataset.
2. Warning threshold ($h^{*}$): A critical value used to define the AD boundary, which usually adopts the empirical formula:
  (17) $$h^{*}=\frac{3(p+1)}{n}$$
  where *p* is the number of descriptors and *n* equals the size of the dataset. When the leverage value of a sample $h_{i}\leq h^{*}$, the sample is determined to be within the AD.

### 4.14. Molecular Docking Evaluation

In drug design, the binding mode and affinity between newly designed compounds and their biological targets are essential indicators of potential biological activity. HIV-1 reverse transcriptase (RT) is a classic target in anti-HIV drug development, characterized by its structural stability and widespread use in NNRTI docking studies [10]. To ensure robust molecular docking results, the crystal structure of HIV-1 RT (PDB ID: **1RT2**) was selected as the docking target. Sybyl-X(2.1.1) software was employed to evaluate the binding affinities [36].

Prior to docking, the reliability of the docking protocol was evaluated by redocking the co-crystallized ligand (TNK) into the NNRTI-binding pocket of HIV-1 RT [37]. The crystallographic ligand was extracted from the protein structure, energy-minimized using the Tripos force field, and subsequently redocked into the prepared binding site using the Surflex-Dock module. The top-ranked docking pose was then superimposed onto the crystallographic binding conformation, and the Root Mean Square Deviation (RMSD) of the heavy atoms was calculated to assess the reliability of the docking parameters and the scoring function. The docking procedure was as follows:

1. Ligand structure preparation: Two-dimensional structures of the novel compounds were drawn in ChemDraw, exported as MOL files, and converted to the MOL2 format using MOE software (version 2007.09).
2. Ligand energy minimization: The MOL2 ligands were imported into Sybyl-X 2.1 and subjected to energy minimization using the Tripos force field to eliminate unstable conformations prior to docking [38].
3. Ligand charge assignment: Atomic charges for the energy-minimized ligands were calculated using the Gasteiger–Hückel method, which assigns partial charges based on atomic electronegativity and the local bonding environment [39].
4. Target protein import: The HIV-1 RT crystal structure file (GZ format) was downloaded from the PDB database and imported into Sybyl-X 2.1 for preprocessing. Residual ligand molecules and crystallographic water molecules were removed to avoid interfering with the binding of new compounds.
5. Molecular docking score calculation: The Surflex-Dock module in Sybyl-X 2.1 was used with the default docking parameters to perform docking and calculate docking scores.

The docking score was used as a qualitative indicator of binding affinity, where higher scores generally suggest stronger predicted interactions [40]. Finally, PyMOL was used to visualize the binding interactions of the novel compound with the highest docking score.

Although the Surflex-Dock Total Score provides a useful empirical measure for ranking ligand–protein binding affinities, it is subject to the inherent limitations of empirical scoring functions. The scoring function is based on simplified energetic terms and does not explicitly account for receptor flexibility, solvent effects, or entropic contributions, which may influence the actual binding affinity. Therefore, the docking scores presented in this study were used primarily as qualitative indicators of binding affinity. To further evaluate the stability and reliability of the predicted binding mode, the novel compound was subsequently subjected to molecular dynamics simulation.

### 4.15. Molecular Dynamics Simulation

To validate the stability of the predicted protein–ligand binding mode under physiological conditions, molecular dynamics (MD) simulations were performed after molecular docking. It should be noted that the MD simulations were conducted solely to evaluate the dynamic behavior and binding stability of the docked complex and were not involved in molecular descriptor generation.

The docked protein–ligand complex was first converted from the PDBQT format to the standard PDB format using PyMOL. Hydrogen atoms were added to the ligand, and saved in MOL2 format, after which Sobtop was employed to generate the corresponding ligand topology files. All MD simulations were performed using the GROMACS 2023.02 simulation package. The Amber99SB-ILDN force field was adopted for the protein, while the TIP3P water model was used to construct an explicit solvent environment. The complex was placed in a periodic simulation box with a minimum distance of 1.2 nm between the protein and the box boundaries to eliminate artifacts arising from periodic boundary conditions. Appropriate numbers of $Na^{+}$ and $Cl^{-}$ ions were then added to neutralize the system and approximate an ionic environment.

Prior to the production simulation, the system was subjected to energy minimization using the steepest descent algorithm followed by the conjugate gradient method to eliminate unfavorable atomic contacts. The minimized system was subsequently equilibrated under both the NVT and NPT ensembles. During the NVT equilibration, position restraints were applied to the solute while the system was gradually heated from 0 K to 300 K. The system was then equilibrated under the NPT ensemble at 300 K and 1 bar to achieve thermodynamic equilibrium. Temperature and pressure were controlled using the V-rescale thermostat and the C-rescale barostat, respectively. The Velocity Verlet algorithm was employed for numerical integration, and the equilibration stage was performed for 100 ps.

Following equilibration, a 100 ns production MD simulation was carried out with a time step of 2 fs, and the complete simulation trajectories were recorded for subsequent analyses. Structural stability and conformational dynamics of the protein–ligand complex were evaluated by calculating the root-mean-square deviation (RMSD), root-mean-square fluctuation (RMSF), radius of gyration ($R_{g}$), solvent-accessible surface area (SASA), and intermolecular hydrogen bonds. Furthermore, the free-energy landscape (FEL) was constructed using RMSD and $R_{g}$ as reaction coordinates. Gibbs free energy was calculated using the built-in g_sham module in GROMACS, and the resulting energy landscapes were visualized as both two-dimensional contour maps and three-dimensional surfaces.

## 5. Conclusions

In conclusion, this study focused on predicting the anti-HIV-1 activity of biphenyl-DAPY-based NNRTI compounds. Multiple QSAR models were systematically constructed and validated. A key contribution of this study is the development of LRNet, a compact architecture tailored to the heuristic descriptor selection framework. In LRNet, the prediction task is represented as a linear baseline supplemented by a nonlinear residual component. This compact architecture is well suited for small datasets, where controlling model complexity is essential to avoid overfitting. Meanwhile, the residual branch allows the model to capture nonlinear effects that cannot be described by the linear component. All models passed the Y-randomization test, indicating that their predictive performance was derived from genuine structure–activity relationships rather than chance correlations. The candidate compounds designed using HM and LRNet showed high predicted activity and favorable binding to **1RT2** in molecular docking analyses. Furthermore, molecular dynamics simulations showed that the designed candidate remained dynamically stable, structurally compatible, and thermodynamically convergent within the binding pocket, supporting the reliability of its predicted binding mode beyond the static docking results. The established QSAR models and analytical framework may provide useful guidance for the structural optimization of biphenyl-DAPY-based NNRTI compounds and the development of highly effective anti-HIV-1 agents.

## Figures and Tables

**Table 2:** Molecular descriptors selected by MI.

Molecular Descriptor Name	Descriptor Abbreviation	MI Score
Kier&Hall index (order 1)	KHI1	0.7385
Min resonance energy for a C-C bond	MREC	0.7028
Avg bond order of a C atom	ABOC	0.6770
Relative number of single bonds	RNSB	0.6715

**Table 3:** Y-randomization results.

Model	Avg $R_{\mathrm{rand}}^{2}$	Avg $Q_{\mathrm{rand}}^{2}$
HM	0.0884	−0.1695
RBF-SVR	0.3453	−0.2430
KRR	0.0925	−0.2799
LMIX-SVR	0.1100	−0.0073
LRNet	0.1853	−0.2239

**Table 5:** Detailed table of Properties of New Compounds.

Compound	Structure	EC_50_ (HM)	EC_50_ (LRNet)	Leverage Value	Docking Score
**M44**	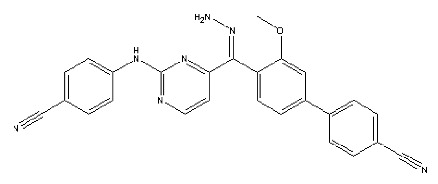	–	–	0.0700	4.7784
**N1**	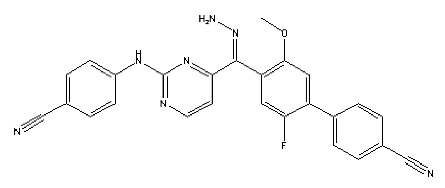	0.0045	0.0031	0.1139	5.7736
**N2**	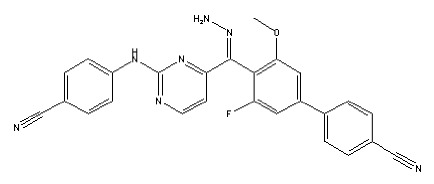	0.0036	0.0022	0.2949	5.0911
**N3**	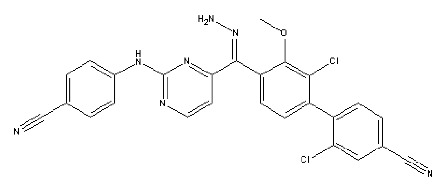	0.0045	0.0030	0.2931	4.5646
**N4**	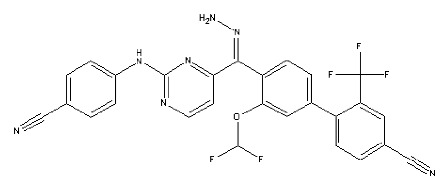	0.0039	0.0028	0.1601	4.4245
**N5**	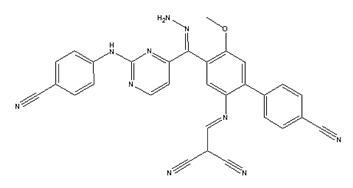	0.0048	0.0049	0.2798	4.7141
**N6**	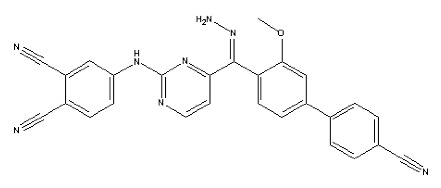	0.0040	0.0030	0.1286	5.4406
**DLV**	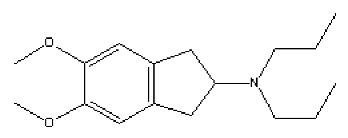	–	–	–	7.6370
**EFV**	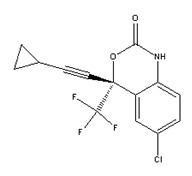	–	–	–	4.8377

**Table 6:** Predicted ADMET and physicochemical properties of the designed derivatives.

Compound	logP	MW	TPSA	SAscore	QED	HIA	AMES Toxicity
**M44**	4.040	445.17	133.00	2.714	0.256	High	Negative
**N1**	4.180	463.16	133.00	2.850	0.244	High	Negative
**N2**	3.990	463.16	133.00	2.853	0.244	High	Negative
**N3**	4.983	513.09	133.00	3.030	0.193	High	Negative
**N4**	5.060	549.13	133.00	3.119	0.126	High	Negative
**N5**	4.619	536.18	192.94	3.541	0.182	High	Positive
**N6**	3.885	470.16	156.79	2.937	0.241	High	Negative

**Table 7:** Performance metrics of different QSAR models.

	R^2^		MSE			
Method	Training Set	Testing Set	Training Set	Testing Set	$Q_{5-\mathrm{fold}}^{2}$	$Q_{\mathrm{LOO}}^{2}$
HM	0.8691 ± 0.0067	0.8973 ± 0.0181	0.0553 ± 0.0031	0.0470 ± 0.0126	0.8460	0.8557
RBF-SVR	0.8511 ± 0.0713	0.7248 ± 0.1915	0.0630 ± 0.00304	0.1226 ± 0.0886	0.8305	0.8457
KRR	0.8481 ± 0.0736	0.7654 ± 0.1257	0.0645 ± 0.0316	0.1045 ± 0.0526	0.8407	0.8447
LMIX-SVR	0.8952 ± 0.0613	0.8943 ± 0.0582	0.0445 ± 0.0268	0.0466 ± 0.0206	0.8349	0.8528
LRNet	0.8838 ± 0.0090	0.9026 ± 0.0187	0.0491 ± 0.0039	0.0448 ± 0.0134	0.8533	0.8541

**Table 8:** Molecular descriptors selected by HM.

Molecular Descriptor Name	Descriptor Abbreviation	Coefficient
Relative number of single bonds	RNSB	−10.940
LUMO energy	LUMO-E	1.8138
Min net atomic charge for a H atom	MNACH	−13.038
Min partial charge for a C atom [Zefirov’s PC]	MPCC	23.821

## Data Availability

The relevant supporting documents and data can be obtained from my repository at https://github.com/whz39coding/public_data/tree/main (accessed on 9 July 2026).

## Abbreviations

NNRTIs	non-nucleoside reverse transcriptase inhibitors
biphenyl-DAPY	biphenyl-diarylpyrimidine
LRNet	Linear Residual Network
QSAR	quantitative structure–activity relationship
MD	Molecular Dynamics

## References

[1] Deeks S.G., Lewin S.R., Havlir D.V. (2013). The end of AIDS: HIV infection as a chronic disease. Lancet.

[2] Katritzky A.R., Lobanov V.S., Karelson M. (1995). QSPR: The correlation and quantitative prediction of chemical and physical properties from structure. Chem. Soc. Rev..

[3] Mackie N., Geretti A.M. (2006). Resistance to non-nucleoside reverse transcriptase inhibitors. Antiretroviral Resistance in Clinical Practice.

[4] Usach I., Melis V., Peris J.E. (2013). Non-nucleoside reverse transcriptase inhibitors: A review on pharmacokinetics, pharmacodynamics, safety and tolerability. J. Int. AIDS Soc..

[5] Dorababu A. (2025). Development of diarylpyrimidine derivatives (& other heterocycles) as HIV-1 and WT RT inhibitors. RSC Med. Chem..

[6] Das K., Bauman J.D., Clark A.D., Frenkel Y.V., Lewi P.J., Shatkin A.J., Hughes S.H., Arnold E. (2008). High-resolution structures of HIV-1 reverse transcriptase/TMC278 complexes: Strategic flexibility explains potency against resistance mutations. Proc. Natl. Acad. Sci. USA.

[7] Pannecouque C., Daelemans D., De Clercq E. (2008). Tetrazolium-based colorimetric assay for the detection of HIV replication inhibitors: Revisited 20 years later. Nat. Protoc..

[8] Cherkasov A., Muratov E.N., Fourches D., Varnek A., Baskin I.I., Cronin M., Dearden J., Gramatica P., Martin Y.C., Todeschini R. (2014). QSAR modeling: Where have you been? Where are you going to?. J. Med. Chem..

[9] Tropsha A. (2010). Best practices for QSAR model development, validation, and exploitation. Mol. Inform..

[10] Hopkins A.L., Ren J., Esnouf R.M., Willcox B.E., Jones E.Y., Ross C., Miyasaka T., Walker R.T., Tanaka H., Stammers D.K. (1996). Complexes of HIV-1 reverse transcriptase with inhibitors of the HEPT series reveal conformational changes relevant to the design of potent non-nucleoside inhibitors. J. Med. Chem..

[11] Chen X.M., Pannecouque C., De Clercq E., Lian Y.X., Corona A., Dettori L., Tramontano E., Wang S., Chen F.E. (2025). Structure-based discovery of novel diarylpyrimidines as potent and selective Non-Nucleoside reverse transcriptase inhibitors: From CH (CN)-Biphenyl-Diarylpyrimidines to CNNH2-Biphenyl-Diarylpyrimidines. Eur. J. Med. Chem..

[12] Prana V., Fayet G., Rotureau P., Adamo C. (2012). Development of validated QSPR models for impact sensitivity of nitroaliphatic compounds. J. Hazard. Mater..

[13] Wicker J.G., Cooper R.I. (2016). Beyond rotatable bond counts: Capturing 3D conformational flexibility in a single descriptor. J. Chem. Inf. Model..

[14] Fukui K. (1982). Role of frontier orbitals in chemical reactions. Science.

[15] Tang T.H., Hu W.J., Yan D.Y., Cui Y.P. (1990). A quantum chemical study on selected *π*-type hydrogen-bonded systems. J. Mol. Struct. THEOCHEM.

[16] Oliferenko A.A., Pisarev S.A., Palyulin V.A., Zefirov N.S. (2006). Atomic charges via electronegativity equalization: Generalizations and perspectives. Adv. Quantum Chem..

[17] Sang Y.L., Pannecouque C., De Clercq E., Wang S., Chen F.E. (2023). Fragment hopping-based design of novel biphenyl-DAPY derivatives as potent non-nucleoside reverse transcriptase inhibitors featuring significantly improved anti-resistance efficacy. J. Med. Chem..

[18] Jin K., Meng G., Chen F.E., Clercq E.D., Pannecouque C. (2017). Discovery of Biphenyl-Substituted Diarylpyrimidines as New Non-Nucleoside HIV-1 Reverse Transcripttase Inhibitors. Proceedings.

[19] Ribone S.R., Leen V., Madrid M., Dehaen W., Daelemans D., Pannecouque C., Brinon M.C. (2012). Synthesis, biological evaluation and molecular modeling of 4, 6-diarylpyrimidines and diarylbenzenes as novel non-nucleosides HIV-1 reverse transcriptase inhibitors. Eur. J. Med. Chem..

[20] Staszewski S., Morales-Ramirez J., Tashima K.T., Rachlis A., Skiest D., Stanford J., Stryker R., Johnson P., Labriola D.F., Farina D. (1999). Efavirenz plus zidovudine and lamivudine, efavirenz plus indinavir, and indinavir plus zidovudine and lamivudine in the treatment of HIV-1 infection in adults. N. Engl. J. Med..

[21] Daina A., Michielin O., Zoete V. (2017). SwissADME: A free web tool to evaluate pharmacokinetics, drug-likeness and medicinal chemistry friendliness of small molecules. Sci. Rep..

[22] Karelson M., Lobanov V.S., Katritzky A.R. (1996). Quantum-chemical descriptors in QSAR/QSPR studies. Chem. Rev..

[23] Stewart J.J. (1990). MOPAC: A semiempirical molecular orbital program. J. Comput.-Aided Mol. Des..

[24] Katritzky A.R., Perumal S., Petrukhin R., Kleinpeter E. (2001). Codessa-based theoretical QSPR model for hydantoin HPLC-RT lipophilicities. J. Chem. Inf. Comput. Sci..

[25] Peng H., Long F., Ding C. (2005). Feature selection based on mutual information criteria of max-dependency, max-relevance, and min-redundancy. IEEE Trans. Pattern Anal. Mach. Intell..

[26] Kraskov A., Stögbauer H., Grassberger P. (2004). Estimating mutual information. Phys. Rev. E—Stat. Nonlinear Soft Matter Phys..

[27] Cortes C., Vapnik V. (1995). Support-vector networks. Mach. Learn..

[28] Williams C.K.I., Rasmussen C.E. (2006). Gaussian Processes for Machine Learning.

[29] Smola A.J., Schölkopf B. (2004). A tutorial on support vector regression. Stat. Comput..

[30] Saunders C., Gammerman A., Vovk V. Ridge regression learning algorithm in dual variables. Proceedings of the Fifteenth International Conference on Machine Learning (ICML).

[31] Gönen M., Alpaydın E. (2011). Multiple kernel learning algorithms. J. Mach. Learn. Res..

[32] Howard A.G., Zhu M., Chen B., Kalenichenko D., Wang W., Weyand T., Andreetto M., Adam H. (2017). MobileNets: Efficient Convolutional Neural Networks for Mobile Vision Applications. arXiv.

[33] Poli R., Kennedy J., Blackwell T. (2007). Particle swarm optimization: An overview. Swarm Intell..

[34] Clerc M., Kennedy J. (2002). The particle swarm-explosion, stability, and convergence in a multidimensional complex space. IEEE Trans. Evol. Comput..

[35] Roy K., Kar S., Ambure P. (2015). On a simple approach for determining applicability domain of QSAR models. Chemom. Intell. Lab. Syst..

[36] Jain A.N. (2007). Surflex-Dock 2.1: Robust performance from ligand energetic modeling, ring flexibility, and knowledge-based search. J. Comput.-Aided Mol. Des..

[37] Vite-Caritino H., Méndez-Lucio O., Reyes H., Cabrera A., Chávez D., Medina-Franco J.L. (2016). Advances in the development of pyridinone derivatives as non-nucleoside reverse transcriptase inhibitors. RSC Adv..

[38] Clark M., Cramer R.D., Van Opdenbosch N. (1989). Validation of the general purpose tripos 5.2 force field. J. Comput. Chem..

[39] Gasteiger J., Marsili M. (1980). Iterative partial equalization of orbital electronegativity—A rapid access to atomic charges. Tetrahedron.

[40] Kitchen D.B., Decornez H., Furr J.R., Bajorath J. (2004). Docking and scoring in virtual screening for drug discovery: Methods and applications. Nat. Rev. Drug Discov..

